# Utilization of Murine Colonoscopy for Orthotopic Implantation of Colorectal Cancer

**DOI:** 10.1371/journal.pone.0028858

**Published:** 2011-12-12

**Authors:** Ehud Zigmond, Zamir Halpern, Eran Elinav, Eli Brazowski, Steffen Jung, Chen Varol

**Affiliations:** 1 The Research Center for Digestive Tract and Liver Diseases, Sourasky Medical Center and the Sackler Faculty of Medicine, Tel-Aviv University, Tel-Aviv, Israel; 2 Department of Immunology, Weizmann Institute of Science, Rehovot, Israel; University of Missouri-Columbia, United States of America

## Abstract

**Background:**

Colorectal-cancer (CRC) research has greatly benefited from the availability of small animal tumor models. Spontaneous and chemically-induced CRC models are widely used yet limited in their resemblance to human disease and are often prolonged, not accurately repetitive, and associated with inflammatory side effects. In-situ murine or human tumor implantation in the gastrointestinal tract of mice is extremely challenging, and limited by inter-animal variability and procedure-related complications and mortality. As a result, in frequent studies CRC is implanted in distal sites, most commonly the subcutaneous region, an approach that is greatly limited by the absence of normal gastrointestinal tumor milieu and has substantial effects on tumor development.

**Aims:**

In this study we aimed to develop a well-tolerated repetitive tool to study CRC in small animals by adapting the murine colonoscopy system to serve as a platform for colonic sub-mucosal orthotopic implantation of human and murine CRC tumor cells.

**Results:**

We report the establishment of a novel small-animal CRC model that is minimally invasive, rapid, well-tolerated, highly reproducible, and confers precise control of tumor number, location and growth rate. Moreover, we show that this model uniquely allows the side-by-side induction of distinct genetically manipulated tumors, enabling the mechanistic study of tumor interaction and cross-talk within the native intestinal microenvironment.

**Conclusions:**

Employment of this new approach may represent a major technical advance for the *in-vivo* study of CRC.

## Introduction

Colorectal-cancer (CRC) is the second leading cause of cancer mortality in many industrialized countries [1]. CRC models in small animals have provided important tools for investigating the underlying etiologic and pathophysiologic mechanisms of CRC development and for the preclinical assessment of novel therapeutic modalities. Numerous murine CRC models have been developed and described in the literature [2], [3]. The main approaches include mutant mice that develop spontaneous tumors, treatment with a wide range of carcinogenic agents, with or without induction of chronic inflammation, and ectopic or orthotopic implantation of tumor cells.

Spontaneous CRC transgenic mouse strains closely imitate human cancer syndromes, such as hereditary nonpolyposis colorectal cancer (HNPCC) [4] and familial adenomatosis polyposis (FAP) [5]. These models have been proven invaluable for the study of the effects of known genetic alterations on the disease course. However, many of these mouse models form adenomas primarily in the small intestine, and are typically associated with a slow growth rate and considerable inter-animal variability [3] The Apc^Min/+^ mouse strain was established based on the results derived from a germ line mutagenesis study with N-ethyl-N-nitrosourea combined with phenotypic screening [5]. Although this model is associated with the appearance of a large number of benign adenomas in the small intestine, CRC tumors develop in less than half of the animals. Nevertheless, treating these mice with the carcinogenic agent azoxymethane (AOM) substantially increases tumor incidence in the colons of these mice, and this model has proven to be useful for studying the effects of chemopreventive compounds [6], [7].

Inducible CRC mouse models, on the other hand, are commonly based on the use of various carcinogenic compounds, such as azoxymethane (AOM), 1,2-dimethylhydrazine (DMH) and others [2]. The chemically induced established tumors share many of the histopathological characteristics of non-hereditary human CRC [2], [8]. Tumor development in these models has been reported to be in the range of 30 weeks. Notably, combination of the carcinogenic compound with chronic inflammation, such as in the case of the AOM/dextran sodium sulfate (DSS) model, has proven to shorten the latency time observed in the classical model to about 10 weeks and to target induction of tumors mainly to the distal colon [8], [9]. These advantages, as well as its high potency, simple mode of application, wide range of susceptibilities, and relative cost efficiency, have made this model very common in CRC research, and an excellent research tool to study early carcinogenic events and to evaluate potential therapeutic approaches [2], [8]. However, in this model tumors develop under the environmental influences of chronic inflammation, a condition that mimics inflammatory bowel disease (IBD)-associated CRC but not the much more common sporadic human CRC. Importantly, all CRC *in- vivo* models mentioned above suffer from an inherent inability to control tumor numbers and growth kinetics.

Several orthotopic murine CRC models have been described, most of which require an operative approach for the injection or implantation of the cancer cells into the colon or cecal layers [10], [11]. The complexity of the surgical procedure, resulting trauma, procedure-related inflammation and mortality, severely limit the value of these systems for the study of innate and adaptive anti-tumor immune responses.

In humans, endoscopic examination of the colon is the most important method for the discovery and follow-up care of CRC. Recently, *in vivo* murine endoscopy has been developed allowing high resolution imaging of the colon in living mice, and enabling researchers to directly visualize and score pathologic tissue changes in the colon [12], [13] and in intra-abdominal organs [14]. Moreover interventions such as repeatable biopsies of colon lesions in living mice during the follow up period of an experiment and their subsequent proteomic analysis have introduced new powerful tools for CRC research [15].

We present here for the first time an adaptation of the murine colonoscopy system and its employment for the establishment of a novel orthotopic mouse model of CRC. This approach provides an easy and straightforward solution to the technical limitations associated with existing CRC models.

## Results

### Establishment of orthotopic mouse model of CRC utilizing the murine colonoscopy system

**Figure 1:**
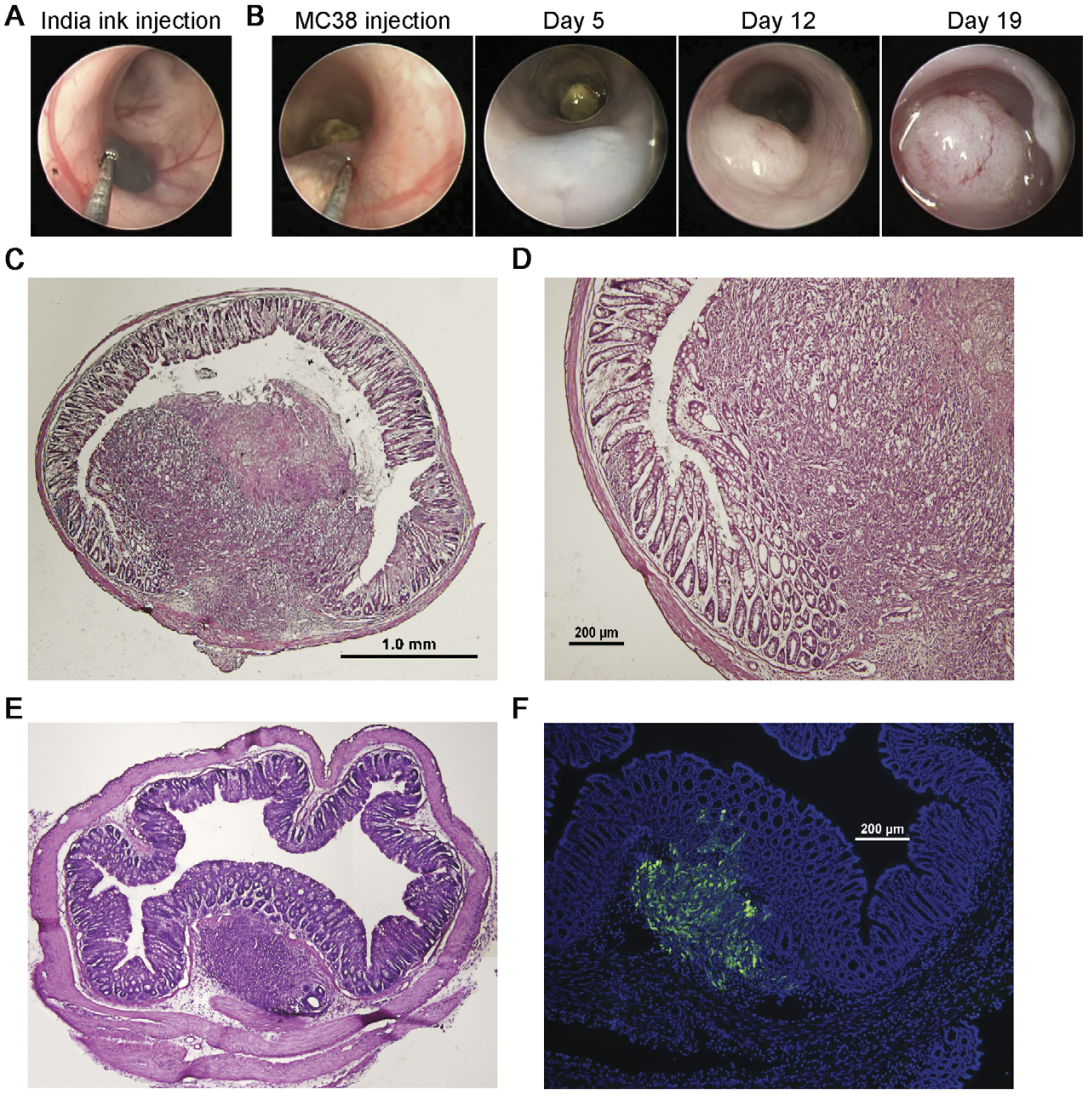
Orthotopic induction of CRC using the mouse colonoscopy system. (A) Colonoscopy image presenting colonic sub-mucosal injection of India-ink dye in C57BL/6 mouse for the assessment of the appropriate injection volume and its distribution at the colonic lamina propria. (B) Representative colonoscopy images indicating the progressive development of CRC tumor within the same C57BL/6 mouse following orthotopic implantation of 1×10^5^ MC38 CRC cells from day 5, through day 12 to day 19. (C–D) Representative image of histology specimen stained with hematoxyline and eosin (H&E) and isolated from colonic injection site at day 14 following injection of 1×10^5^ MC38 CRC cells. Magnifications: C- [x40], D – [x100]. Note the boundary area between the tumor and the adjacent normal mucosa and the projection of the tumor through the epithelial layer into the lumen. (E) Representative image showing H&E staining of histology specimen of murine-CRC tumor generated 5 days following orthotopic implantation of MC38 CRC tumor cells into C57BL/6 mice (Magnification x40). (F) Fluorescent microscopic image showing GFP labeled tumor cells and their dissemination through the epithelial layer toward the lumen (Magnification x100). Data are representative of three independent experiments.

Small animal endoscopy is considered today as the gold standard tool for the surveillance of colonic inflammatory disorders and CRC. In order to adapt this system for the purpose of orthotopic implantation of CRC tumor cells into the colonic sub-mucosa we have specially designed a hypodermic needle that can pass through the endoscopic sheath working channel of the Karl Storz Coloview miniendoscopic system. The hypodermic needles were custom made according to our specification by Cadence Inc. U.S.A., and are made from 8 inch long flexible stainless flexible steel, with 30 gauge outer diameters, and a short bevel at a 45 degree angel (**Figure S1**). This hypodermic needle confers several design advantages; the flexible steel together with needle's dimensions alleviate the injection maneuver and allow the smooth pass through the working channel and the Luer lock, which is screwed on it to avoid air leakage. Moreover, the blunt bevel allows injection into the sub-mucosa with reduced risk for perforation or spilling of the injected material to the lumen. We first examined the tissue distribution of injected reagents and assessed the appropriate injection volume by injecting India ink marking dye into the colonic sub-mucosa layer. An injection volume of 50 µl gave the best combination of minimal leakage and focal scattering of the ink beneath the epithelial layer (**Figure 1A**). Flushing of the colonic lumen by applying saline through the examination sheath of the endoscope confirmed the localization of the injected ink beneath the epithelial layer (**Movie S1**). Histological analysis confirmed that the India ink is retained within the colonic lamina propria even 5 days following its orthotopic injection (**Figure S2A**). Importantly, histological analysis of mice injected with sterile Dulbecco's Phosphate Buffered Saline without calcium and without magnesium (PBS^-/-^) revealed a normal healthy colonic architecture at the injection site confirming that this method is minimally invasive (**Figure S2B**).

The MC-38 murine colon tumor is a grade III adenocarcinoma, which was chemically induced in a female C57BL/6 mouse and used since then as a transplantable mouse tumor model [16]. To test the feasibility of the orthotopic tumor implantation method, we injected 1×10^5^ MC38 CRC tumor cells into the colonic sub-mucosa of syngeneic C57BL/6 mice. Repetitive colonoscopy of recipient mice revealed the progressive rapid development of the tumor breaking into the gut lumen specifically at the injection site. Endoscopic grading according to tumor size relatively to the circumference of the colon,as established by Becker et al [12], revealed progressive tumor growth from grade 1 at day 5 to grade 4 by day 12 and reaching grade 5, occupying almost the entire colonic circumference, by only day 19 (**Figure 1B**, **and Movie S2**). Hematoxyline and eosin (H&E) staining of histology sections revealed a typical CRC architecture of the tumors with normal adjacent mucosa and the projection of the tumor through the epithelial layer into the lumen (**Figure 1C, D**). Histological analysis of colonic tissue isolated from mice subjected to orthotopic implantation of 1×10^5^ MC38 CRC cells expressing GFP confirmed the establishment of a small tumor within the lamina propria and the penetration of tumor derived GFP^+^ cells through the epithelial layer already by day 5 (**Figure 1E, F**). Using this method, we were also able to efficiently induce the formation of human CRC tumors by implanting the human CRC cell-lines SW620, SW480, LS174T and HT-29 into immuno-deficient NUDE or NOD/SCID recipient mice (**Figure S3A**). Pathological assessment of orthotopically established human CRC tumors using this model have confirmed high similarity to CRC tumors isolated from human patients (**Figure S3B**). Specifically, human CRC tumors formed by the orthotopic implantation of LS174T and HT29 cells showed histological features characteristic of colonic differentiated adenocarcinoma such as glandular structures lined with neoplastic epithelium containing goblet cells and luminal necrotic material, while the SW620 human CRC cells formed a tumor morphology typical of poorly to undifferentiated carcinoma with nested and diffused patterns and lack of tubular formation (**Figure S3B**). Interestingly, both HT29 and LS174T human CRC cell lines were formed from a primary adenocarcinoma of the colon, while the SW620 was established from a lymph node metastasis.

**Figure 2:**
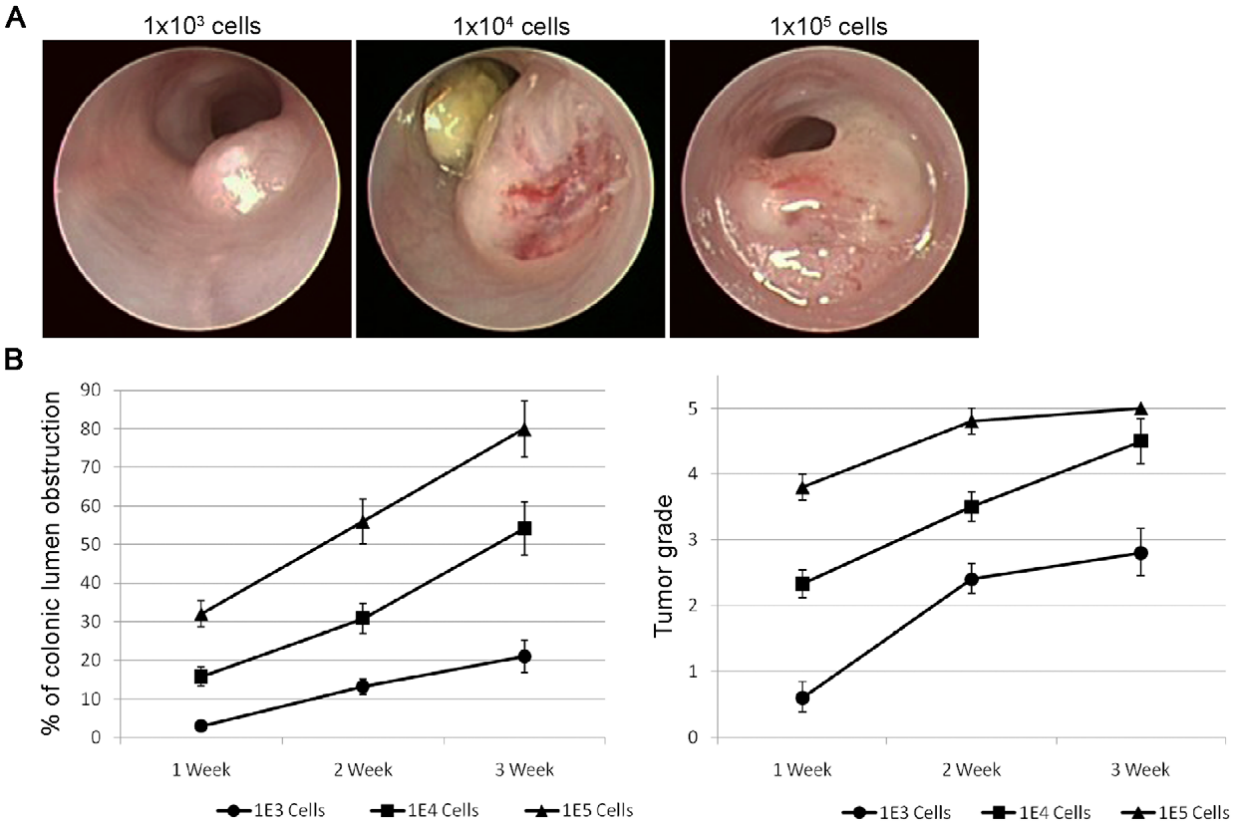
Absolute control on growth kinetics of orthotopically induced tumors. (A) Representative colonoscopy images demonstrating the correlation between amount of injected MC38 tumor cells and the size of the established CRC tumor 3 weeks following orthotopic tumor cell implantation. (B) Graphs summarizing the growth kinetics, circumference percentage and developmental grade of colonic tumor in correlation to the amount of injected MC38 CRC cells. Note the rapid establishment of grade 5 tumors already 2 weeks following implantation of only 1×10^5^ MC38-CRC cells. Each group consisted of 5 mice.

In order to evaluate the growth kinetics of the CRC tumors using this method we injected C57BL/6 mice with distinct amounts of MC38 cells (10E3, 10E4 and 10E5 cells) and followed the colonic circumference and developmental grade of established tumors over time by colonoscopy (**Figure 2A**). The results graphically summarized in **figure 2B** show that the tumor growth kinetics was in direct correlation to the amount of injected cells reaching colon circumference of 21% (+/−4.22), 54.1% (+/−7.0), and 80% (+/−7.2) 3 weeks following the injection of 10^3^, 10^4^, and 10^5^ MC38 cells, respectively. Statistical analysis comparing each time point (1,2 and 3 weeks) for the different groups (10E3, 10E4 and 10E5 cells) confirmed significant differences (at least p<0.05) in all the time points for percentage of colonic circumference as well as for tumor grade. Exceptional was the comparison of tumor grade between the 10E4 &10E5 groups at week 3 as most of the tumors in these groups were already grade 5 that includes all tumors above 50% of colonic circumference.

Collectively, we established here a new method for the efficient orthotopic implantation of CRC tumors using murine endoscopy in a rapid and minimal invasive manner, with a limited amount of cells required for injection, and with an absolute control on tumor's incidence, location and growth kinetics.

### Orthotopic implantation of genetically manipulated CRC-tumor cells

**Figure 3:**
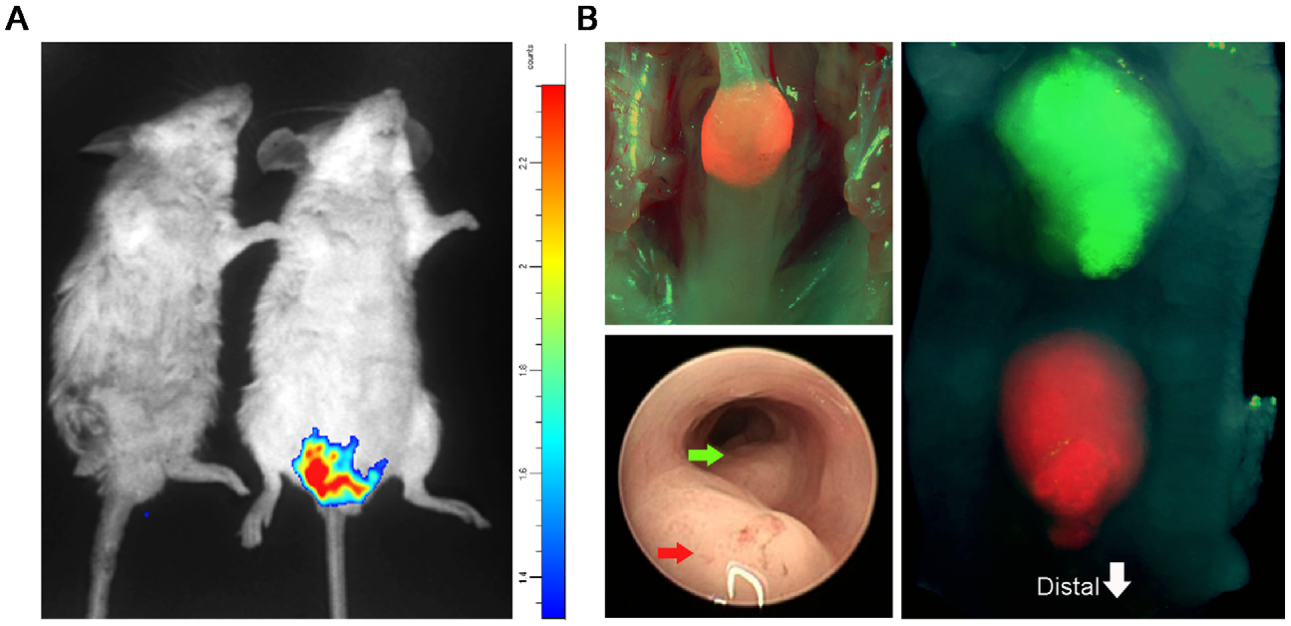
Orthotopic induction of distinct genetically manipulated CRC tumors. (A) Color-coded representative image of whole body bioluminescence optical imaging of BALB/c mice 2 weeks following sub-mucosal injection of PBS^-/-^ (left mouse) or 1×10^5^ CT26 CRC tumor cells expressing luciferase and 10 minutes following i.p injection of D-Luciferin. Note the luminescence emission specifically from the area of injected CRC tumor cells (right mouse). (B) Upper left panel – fluorescent stereo-microscopic image overlaid on a photo graphic image of recipient C57BL/6 mouse colon 3 weeks following its orthotopic injection with MC38 CRC cells that were genetically manipulated by lentiviral reporter system to express RFP, magnification x10. Down left panel – colonoscopy image of C57BL/6 mouse colon following orthotopic implantation of 2 adjacent MC38 CRC tumor cells; MC38-RFP (red arrow) and MC38-GFP (green arrow). Right panel - fluorescent stereo-microscopic image overlaid on a photo graphic image of recipient C57BL/6 mouse opened colon 2 weeks following side-by-side orthotopic injection of MC38-RFP (distal) and MC38-GFP (proximal) CRC tumor cells, magnification x10. Note the induction of 2 adjacent CRC tumors that differ only by the expression of the transduced reporter gene. Data are representative of three independent experiments.

One of the benefits of cancer implantation over spontaneous developing tumor models is the ability to genetically modify the established tumor. As a proof of principle, we injected BALB/c mice with 1×10^5^ CT26 murine CRC cells, which had previously been genetically manipulated to express luciferase. Whole body bioluminescence optical imaging (Biospace Photon imager) revealed the existence of a CRC tumor specifically around the injection site that could be further semi-quantified according to its emitted luminescence (**Figure 3A**). Notably, this approach may be useful for the assessment of tumor burdens without the need to sacrifice the mice. An additional genuine advantage of the endoscopic implantation model is the capability to implant several adjacent yet distinct tumors in the same mouse, which were genetically modified to over-express or silence genes of interest, enabling their direct comparison in an identical host milieu. Using a lentiviral reporter system, we transduced two distinct fluorescent reporter genes into the MC38 cell line to establish the MC38-GFP and MC38-RFP lines (**Figure 3B** **upper left panel**). Injection of the MC38-GFP cells and adjacent to them the MC38-RFP cells resulted in the co-formation of two genetically identical tumors that vary only by their location and expression of the inserted reporter genes (**Figure 3B** **lower left panel and right panel**).

## Discussion

We describe herein a novel orthotopic murine CRC model based on the adaptation of the commercially available miniendoscopy system that confers many advantages compared to known murine CRC models. It is an easy to employ, minimally invasive, and highly reproducible procedure that can be used on mice from any genetic background. In this model both murine and human CRC-tumors can be induced. The model allows also precise control of tumor incidence and location within the colon. In addition, this method enables absolute control on tumor growth kinetics; it can be very rapid reaching grade 5 in 2 weeks following the implantation of only 1×10^5^ tumor cells, or one can slow it by injecting low number of cells. Importantly, this approach avoids collateral damage and inflammation seen in many of the other currently employed models. Moreover, it uniquely enables the induction of genetically manipulated CRC-tumors and the comparison of several distinct tumors established side-by-side in the same mouse avoiding the inter-animal variability.

Ectopic subcutaneous implantation of tumor cell lines is widely used as a research tool, especially for the assessment of cytostatic therapeutic interventions [17]. However, ectopic implantation disregards the complexity of the unique characteristic makeup of the native microenvironment. Indeed, it has been well established that the incongruent host stroma can affect tumorigenicity and cell cycle regulation compared with implantation in the native tissue bed [18], [19]. The advantage of choosing an orthotopic approach is of particularly importance for the intestinal milieu, which is the home of unique immunological cellular and molecular entities that resides in continuous interaction with the microbiota and constitutive exposure to dietary antigens.

Performance of more then 200 orthotopic CRC implantations utilizing this method has ensured that this model is well-tolerated and highly reproducible. Yet, a possible complication of the procedure is perforation of the colon and potentially leakage of air and cells to the peritoneal cavity. Currently, in our hands, this complication is rare, constituting less than 5 percent of all procedures. Mortality during the procedure is extremely rare and can happen due to the anesthesia or perforation. Tumor incidence in surviving mice (95%) is 100%, always culminating in a sole tumor development specifically at the injection site. Of note, the use of a rigid scope allows access only to the distal half of the colon and thus, restricts the orthotopic implantation by this approach to this area.

CRC is still the second leading cause of cancer mortality in many industrialized countries. Experiments done in small animals have proven to be essential to the understanding of the pathophysiology of the disease as well as for the preclinical assessment of novel therapeutic modalities. Nevertheless, murine CRC models are limited in their similarity to human disease. This is especially true concerning the formation of CRC tumor metastases. One notable disadvantage of CRC murine models is the general lack of invasive and metastatic phenotype [2]. In the AOM/DSS model infiltrative cords of epithelial cells were described to breach the muscularis mucosa and reach also the sub-mucosal lymphatic system, although very infrequently [20]. Similarly, we seldom witness local invasiveness of tumor-derived cells, but never detect distal metastases using our approach. The relatively rapid growth kinetics associated with our model, which coerces the early sacrifice of implanted mice caring a grade 5 CRC tumor, is probably becoming a disadvantage for induction of a metastatic tumor. Indeed, even orthotopic implantation of human CRC cells, known to be aggressively metastatic, failed to induce a metastatic tumor. The common hindrance shared by murine CRC models to establish a metastatic tumor may imply that the intestinal murine milieu is not supportive of metastases formation. Nevertheless, this can be turn into a desired model system for those who wish to investigate the potential role of malignant genes. In support of this, our system can be utilized to induce the formation of genetically manipulated tumors in which we over-express or silence genes of interest.

We believe that this new orthotopic CRC model represents a major technical advance for the *in-vivo* study of CRC, and can be particularly used to investigate new therapeutic modalities, tumor's genetics and growth mechanisms, and its interactions in the context of the native intestinal microenvironment.

## Methods

### Ethics Statement

All animal studies were approved by the Tel Aviv Sourasky Medical center ethical committee for animal studies and conformed to the highest international standards of humane care of animals in biomedical research. Committee approval number – 037_b3428_8.

### Animals

8–10 week-old males C57BL/6, Balb C and Athymic nude mice were obtained from Harlan biotech (Rehovot, Israel). 8–10 week-old males NOD SCID mice were kindly provided by prof. Tsvee Lapidot (Weizmann Institute of Science, Rehovot, Israel).

Athymic nude mice were sub-lethaly irradiated (450 rad) before the orthotopic implantation of human CRC cell lines to eliminate immuno-rejection. Animals had unrestricted access to food and water, were housed in temperature and humidity-controlled rooms, and were kept on a 12-hour light/dark cycle.

### Colo-Rectal tumor cells

Murine C57BL/6 CRC tumor cells (MC38) were kindly provided by Dr. Avi Eisenthal (Tel Aviv Sourasky Medical Center). Luciferase stably- transfected murine CRC tumor cells (CT26) were kindly provided by Prof. Lea Eisenbach (Weizmann Institute of Science Rehovot, Israel). The human CRC tumor cell-lines SW620, SW480 and LS174T were kindly provided by prof. Zelig Eshhar (Weizmann Institute of Science, Rehovot, Israel). The HT-29 human CRC cell-line was the generous gift of Dr. Isabel Zvibel (Tel-Aviv Sourasky Medical Center, Tel-Aviv, Israel). All cell lines were maintained in 5% CO_2_ at 37°C in high glucose DMEM medium supplemented with: 10% fetal bovine serum, 1% of Penicillin streptomycin antibiotic solution, 1% L-glutamin solution, and 1% sodium pyruvate solution.

### Generation of MC38 CRC tumor cells expressing GFP or RFP

MC38-GFP and MC38-RFP CRC tumor cells were generated by transduction with pCSC-SP-PW-IRES-GFP/RFP lentiviruses designed to express GFP or RFP with M.O.I of 50 (kindly provided by Dr. Alon Chen, Weizmann Institute of Science Rehovot, Israel). Subsequently, MC38 cells expressing GFP or RFP were sorted to high purity using a high speed FACSAria II sorter (Beckton-Dickinson).

### Murine endoscopic system

We employed a high resolution mouse video endoscopic system (‘‘Coloview system’’) previously described for murine endoscopic procedures [12]–[14] which consists of a miniature endoscope (scope 1.9 mm outer diameter), a xenon light source, a triple chip camera, and an air pump to achieve regulated inflation of the mouse bowel (Karl Storz, Tuttlingen, Germany). The endoscopic procedure was viewed on a color monitor and digitally recorded on tape.

### Orthotopic colonic sub-mucosal implantation of CRC cells

Mice were anesthetized using Ketamine/Xylazine. Sub-mucosal injections were accomplished using stainless flexible stainless steel; 8 inch long, 30 gauge and 45 degree bevel hypodermic needles custom made according to our specification (Cadence Inc. U.S.A). The needle was inserted through Luer lock (Söllner, GmbH) screwed on the working channel of the scope to avoid air leakage. Subsequently, the scope was inserted into the mouse colon and following its inflation the needle was brought through the working channel to the scope's front. The CRC cell implantation procedure was performed by two coordinated persons; one person was navigating the colonoscopy while the other person was operating the injection maneuver. The injection was performed under observation by a very gentle sub-mucosal penetration with the open side of the bevel heading up in a flat angle. A volume of 50 microliter CRC tumors cells was then injected into the colonic sub-mucosa.

### *In-vivo* imaging

To follow luciferase-expressing CT26 CRC tumor implantation and growth in BALB/c mice, 50 µl D-Luciferin (30 µg/ml) was i.p injected 10 minutes before and subsequently mice were inserted into the closed dark chamber of the whole body cooled CCD camera Photon Imager system (Biospace Lab, France). For *in-vivo* visualization of GFP and RFP positive tumors Leica MZ16F fluorescent stereomicroscopy system was used (Leica Microsystems, Germany).

### Histology

Tissues were fixed in 4% paraformaldehyde overnight at 4°C, embedded in paraffin, serially sectioned (15 µm), and stained with hematoxylin and eosin (Sigma).

Slides were observed using the Olympus BX51 microscope, and image acquisition was conducted with the Olympus DP70 camera and DP-Manager software.

### CRC tumor growth kinetics

C57BL/6 mice were orthotopically sub-mucosal injected with 10^3^, 10^4^, or 10^5^ MC38 cells. The tumor's colonic circumference and developmental stage were monitored over time by colonoscopy every week for 3 weeks following cell implantation. Endoscopic grading was done according to tumor size relatively to the circumference of the colon as established by Becker et al [12]. In short, tumor size was graded as follows: Grade 1 (just detectable tumor), Grade 2 (tumor covering up to 1/8 of colonic circumference), Grade 3 (tumor covering up to 1/4 of the colonic circumference), Grade 4 (tumor covering up to 1/2 of the colonic circumference) and Grade 5 (tumor covering more than 1/2 of the colonic circumference).

### Statistical Analysis

The results were analyzed by one-way ANOVA and post analysis with Bonferroni's multiple comparison tests.

## Supporting Information

Figure S1 **Specially customized hypodermic needles used to orthotopically inject the tumor cells into the colonic sub-mucosa.** The hypodermic needles are made from 8 inch long flexible stainless steel, with 30 gauge outer diameters, and a short bevel at a 45 degree angel. Note that the needle is inserted through the Luer lock that is subsequently screwed on the working channel of the endoscope to avoid air leakage.
(TIF)

Figure S2 **Sub-mucosal injections of PBS^-/-^ and India ink.** Representative images of histology specimens stained with hematoxyline and eosin (H&E) and isolated from colonic injection site at day 5 following injection of 50 µl of (A) India ink (Magnification x40), (B) India ink (Magnification x100), (C) PBS^-/-^, note the normal healthy architecture of the colon. Data are representative of three independent experiments.
(TIF)

Figure S3 **Orthotopic induction of human CRC tumors in immuno-deficient mice.** (A) Representative images of histology specimens stained with hematoxyline and eosin (H&E) and isolated at day 35 following orthotopic injection (2×10^5^ cells each) of the human CRC cell lines: SW620, SW480, LS174T and HT29 into NOD/SCID or sub-lethally irradiated NUDE mice. Data are representative of three independent experiments. (B) Histo-pathological comparison between human CRC tumors established by the orthotopic implantation of SW620, HT29, and LS174T into immuno-deficient mice and human colorectal adenocarcinoma.
(TIF)

Movie S1 **Endoscopic live imaging of sub-mucosal injection of India ink dye.** Representative endoscopic video imaging demonstrating the procedure of India-ink dye injection into the colonic sub-mucosa of C57BL/6 mice using the mouse colonoscopy system. Note the focal sub-epithelial distribution of injected dye and its remaining following the colonic flushing.
(WMV)

Movie S2 **Endoscopic live imaging of the progressive localized growth of a CRC-tumor following its orthotopic implantation.** Endoscopic video imaging showing the orthotopic implantation of CRC-tumor cells and the progressive growth of the resulted tumor, specifically at the injection site and at different time points within the same recipient.
(WMV)
