# Supplementary figures and images for: Utilization of Murine Colonoscopy for Orthotopic Implantation of Colorectal Cancer
Source: PLoS One. 2011 Dec 12;6(12):e28858. doi: 10.1371/journal.pone.0028858 (PMC3236220; doi:10.1371/journal.pone.0028858)

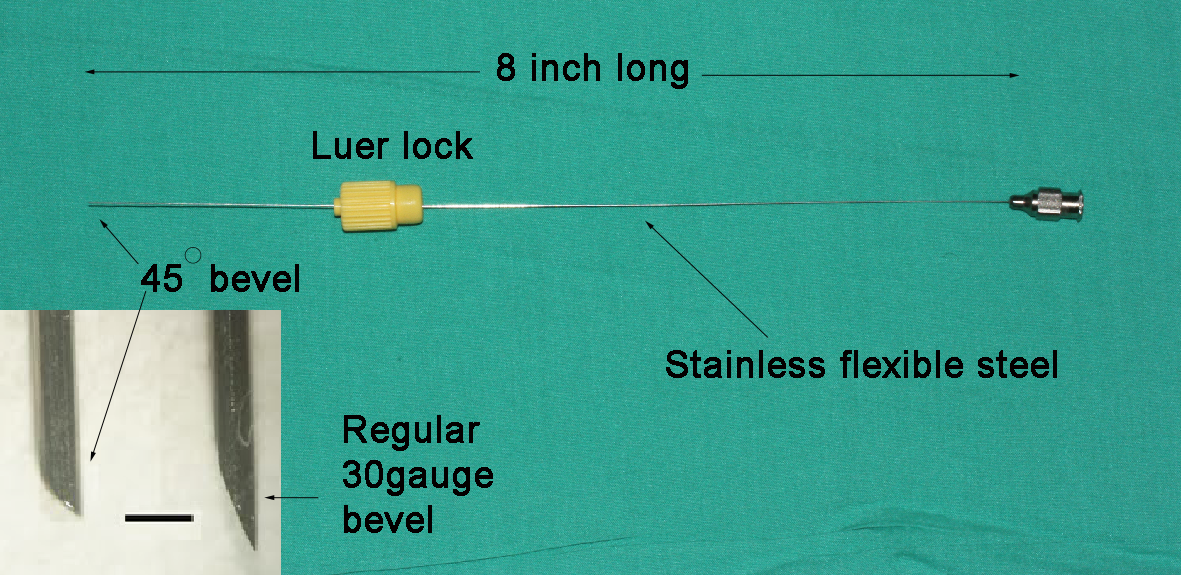

Supplement: Figure S1 — Specially customized hypodermic needles used to orthotopically inject the tumor cells into the colonic sub-mucosa. The hypodermic needles are made from 8 inch long flexible stainless steel, with 30 gauge outer diameters, and a short bevel at a 45 degree angel. Note that the needle is inserted through the Luer lock that is subsequently screwed on the working channel of the endoscope to avoid air leakage. (TIF) [file pone.0028858.s001.tif]

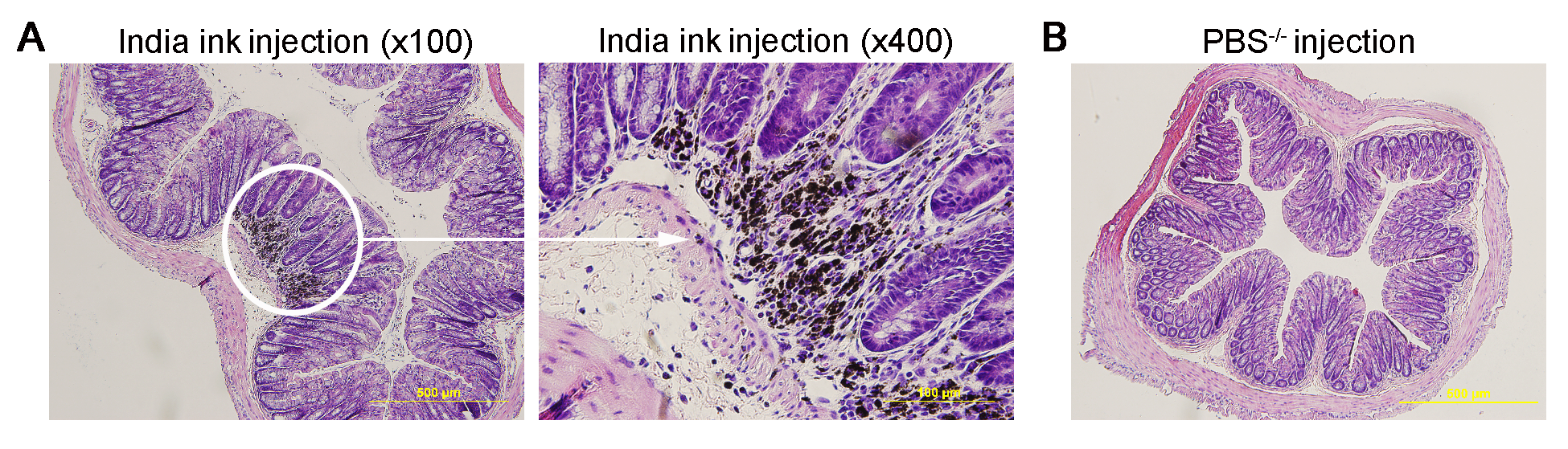

Supplement: Figure S2 — Sub-mucosal injections of PBS-/- and India ink. Representative images of histology specimens stained with hematoxyline and eosin (H&E) and isolated from colonic injection site at day 5 following injection of 50 µl of (A) India ink (Magnification x40), (B) India ink (Magnification x100), (C) PBS-/-, note the normal healthy architecture of the colon. Data are representative of three independent experiments. (TIF) [file pone.0028858.s002.tif]

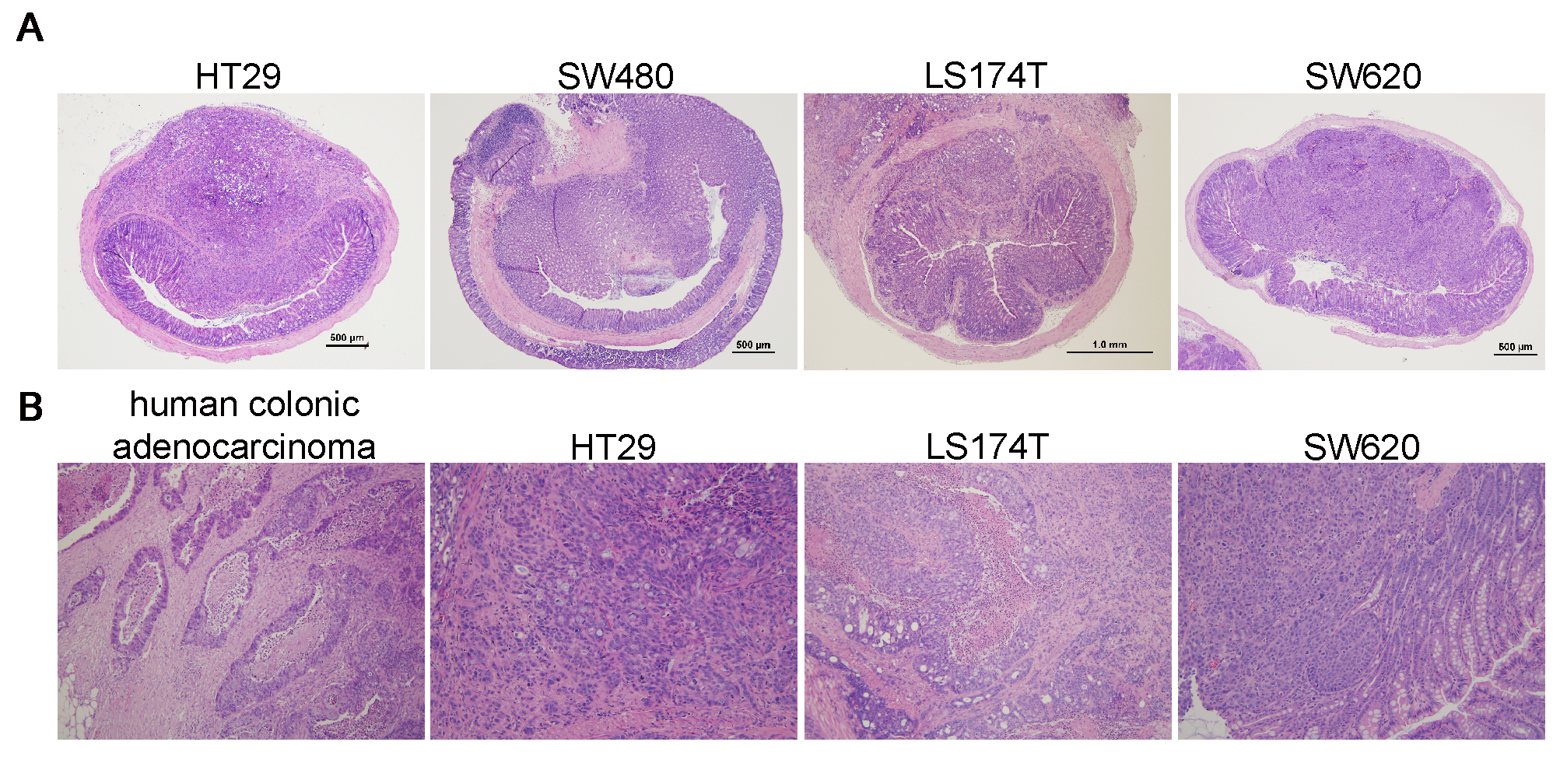

Supplement: Figure S3 — Orthotopic induction of human CRC tumors in immuno-deficient mice. (A) Representative images of histology specimens stained with hematoxyline and eosin (H&E) and isolated at day 35 following orthotopic injection (2×10^5^ cells each) of the human CRC cell lines: SW620, SW480, LS174T and HT29 into NOD/SCID or sub-lethally irradiated NUDE mice. Data are representative of three independent experiments. (B) Histo-pathological comparison between human CRC tumors established by the orthotopic implantation of SW620, HT29, and LS174T into immuno-deficient mice and human colorectal adenocarcinoma. (TIF) [file pone.0028858.s003.tif]
